# Supplementary material for: 4-hexylresorcinol-induced protein expression changes in human umbilical cord vein endothelial cells as determined by immunoprecipitation high-performance liquid chromatography
Source: PLoS One. 2020 Dec 15;15(12):e0243975. doi: 10.1371/journal.pone.0243975 (PMC7737996; doi:10.1371/journal.pone.0243975)
Supplement: S1 Data — (DOCX) [file pone.0243975.s001.docx]

**S1 Data**

**Mathematical algorithm for IP-HPLC analysis**

- IP-HPLC peak area (mAU*s) contains antigen peak ({Ag}), antibody peak ({Ab}), and antigen-antibody complex peak ({Ag-Ab}).

A _(mAU*s)_ = {Ag_1_} + {Ab_1_} + {Ag_2_-Ab_2_}

- UV spectrum shows relatively proportional value (α) between ({Ag} + {Ab}) and {Ag-Ab}; {Ag-Ab} = α({Ag} + {Ab})

A _(mAU*s)_ = {Ag_1_} + {Ab_1_} + α({Ag_2_} + {Ab_2_})

A _(mAU*s)_ = ({Ag_1_} + α({Ag_2_}) +({Ab_1_} + α{Ab_2_})

{Ag_1_} + α({Ag_2_} _(mAU*s)_ = A - ({Ab_1_} + α{Ab_2_})

- When the antibody was monospecific or monoclonal to antigen, Ag1/Ag2 = Ab1/Ab2 = β.

{Ag_1_} + $\frac{\alpha}{\beta}$({Ag_2_} _(mAU*s)_ = A - ({Ab_1_} + α{Ab_2_})

{Ag_1_} (1 + $\frac{\alpha}{\beta}$) _(mAU*s)_ = A - ({Ab_1_} + α{Ab_2_})

{Ag_1_} _(mAU*s)_ = $\frac{\beta(A - \left( \left\{ \mathrm{Ab}1 \right\}+ \alpha\left\{ \mathrm{Ab}2 \right\} \right))}{\alpha+\beta}$

And

{Ag_1_} + α({Ag_2_} _(mAU*s)_ = A - ({Ab_1_} + α{Ab_2_})

β({Ag_2_} + α({Ag_2_} _(mAU*s)_ = A - ({Ab_1_} + α{Ab_2_})

β({Ag_2_} + α({Ag_2_} _(mAU*s)_ = A - ({Ab_1_} + α{Ab_2_})

{Ag_2_}(α + β) _(mAU*s)_ = A - ({Ab_1_} + α{Ab_2_})

{Ag_2_}(α + β) _(mAU*s)_ = $\frac{A - (\{Ab1\} + \alpha\{Ab2\})}{\alpha+ \beta}$

- And then, the objective antigen expression was {Ag_1_} + {Ag_2_}.

{Ag_1_} + {Ag_2_} _(mAU*s)_ = $\frac{\left( 1+\beta\right)A-2(\left\{ Ab1 \right\}+ \alpha\left\{ Ab1 \right\})}{\alpha+ \beta}$

- {Ag_1c_} + {Ag_2c_} _(mAU*s)_ is an objective antigen expression of control group, while {Ag_1e_} + {Ag_2e_} _(mAU*s)_ is an objective antigen expression of experimental group.

- The ratio compared between experiment and control objective antigen expression is ({Ag_1e_} + {Ag_2e_})/({Ag_1c_} + {Ag_2c_}).

$\frac{\{Ag1e\} + \{Ag2e\}}{\{Ag1c\} + \{Ag2c\}}$ = $\frac{Ae\left( 1+\beta\right) - 2(\left\{ Ab1e \right\} + \alpha\left\{ Ab2e \right\})}{Ac\left( 1+\beta\right) - 2(\left\{ Ab1c \right\} + \alpha\left\{ Ab2c \right\})}$

= $\frac{Ae - \frac{2(\left\{ Ab1e \right\} + \alpha\left\{ Ab2e \right\}}{1 + \beta}}{Ac - \frac{2(\left\{ Ab1c \right\} + \alpha\left\{ Ab2c \right\}}{1 + \beta}}$

- $\frac{2(\left\{ Ab1e \right\} + \alpha\left\{ Ab2e \right\}}{1 + \beta}$ and $\frac{2(\left\{ Ab1c \right\} + \alpha\left\{ Ab2c \right\}}{1 + \beta}$ are replaceable with γA_ave_ (A_ave_ is average of A_c_ and A_e_).

- Therefore,

$\frac{\{Ag1e\} + \{Ag2e\}}{\{Ag1c\} + \{Ag2c\}}$ = $\frac{Ae - \gamma Aave}{Ac - \gamma Aave}$

- Because ({Ag_1e_} + {Ag_2e_}) and ({Ag_1c_} + {Ag_2c_}) are mathematically hypothetical value (mAU*s), their square root value may approximate the comparable expression level (mAU).

$\sqrt{\frac{\{Ag1e\} + \{Ag2e\}}{\{Ag1c\} + \{Ag2c\}}}$ = $\sqrt{\frac{Ae - \gamma Aave}{Ac - \gamma Aave}}$

$\frac{Experiment antigen expression level}{Control antigen expression level}$ = $\sqrt{\frac{Ae - \gamma Aave}{Ac - \gamma Aave}}$ x 100 (%)

- γ can be determined by experimental IP-HPLC. If 15%-reduced amount of objective protein sample was applied to Protein A/G bead column compared to control group. The IP-HPLC results were as follow;


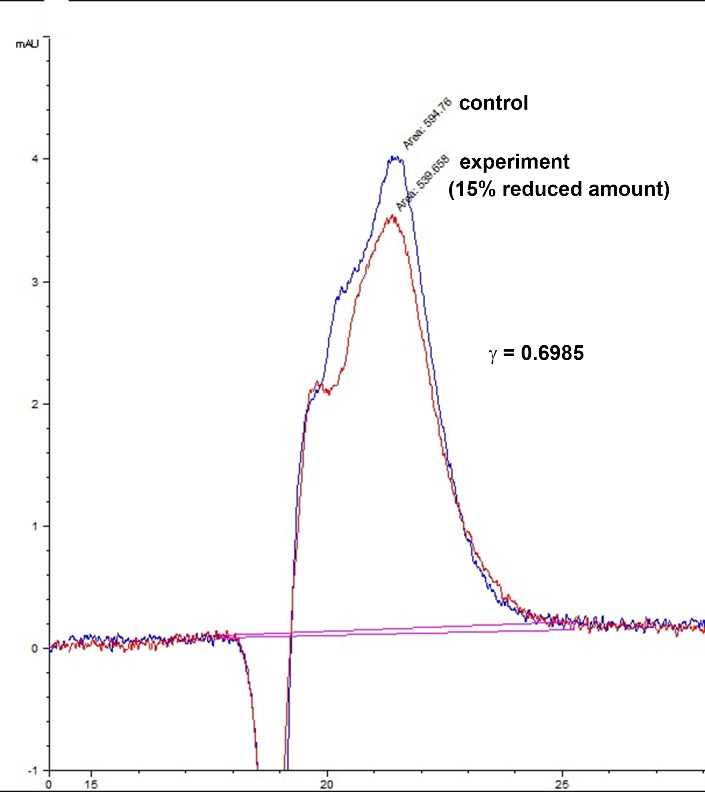


When the eluted proteins containing antibody were analyzed with 30 cm long column at low running speed (0.3 mL/min), the proteins were slightly separated but still appeared much overlapped in chromatography.

- And then,

$\frac{Experiment antigen expression level}{Control antigen expression level}$ = $\sqrt{\frac{Ae - \gamma Aave}{Ac - \gamma Aave}}$ x 100 = 85 (%)

$\sqrt{\frac{Ae - \gamma Aave}{Ac - \gamma Aave}}$ x 100 = 85 (%)

$\sqrt{\frac{539.658 - 567.209\gamma}{594.76 - 567.209\gamma}}$ x 100 = 85 (%)

Therefore, constant γ can be calculated as 0.6985, and used to subtract other elements besides objective protein.

From this algorithm, the relative ratio (%) between objective protein level and control protein level can be obtained, albeit it is impossible to get the concentration of objective protein through IP-HPLC.
