# Supplementary material for: 4-hexylresorcinol-induced protein expression changes in human umbilical cord vein endothelial cells as determined by immunoprecipitation high-performance liquid chromatography
Source: PLoS One. 2020 Dec 15;15(12):e0243975. doi: 10.1371/journal.pone.0243975 (PMC7737996; doi:10.1371/journal.pone.0243975)
Supplement: S2 Data — (DOCX) [file pone.0243975.s002.docx]

**S2 Data**

**Representative chromatography through IP-HPLC analysis**


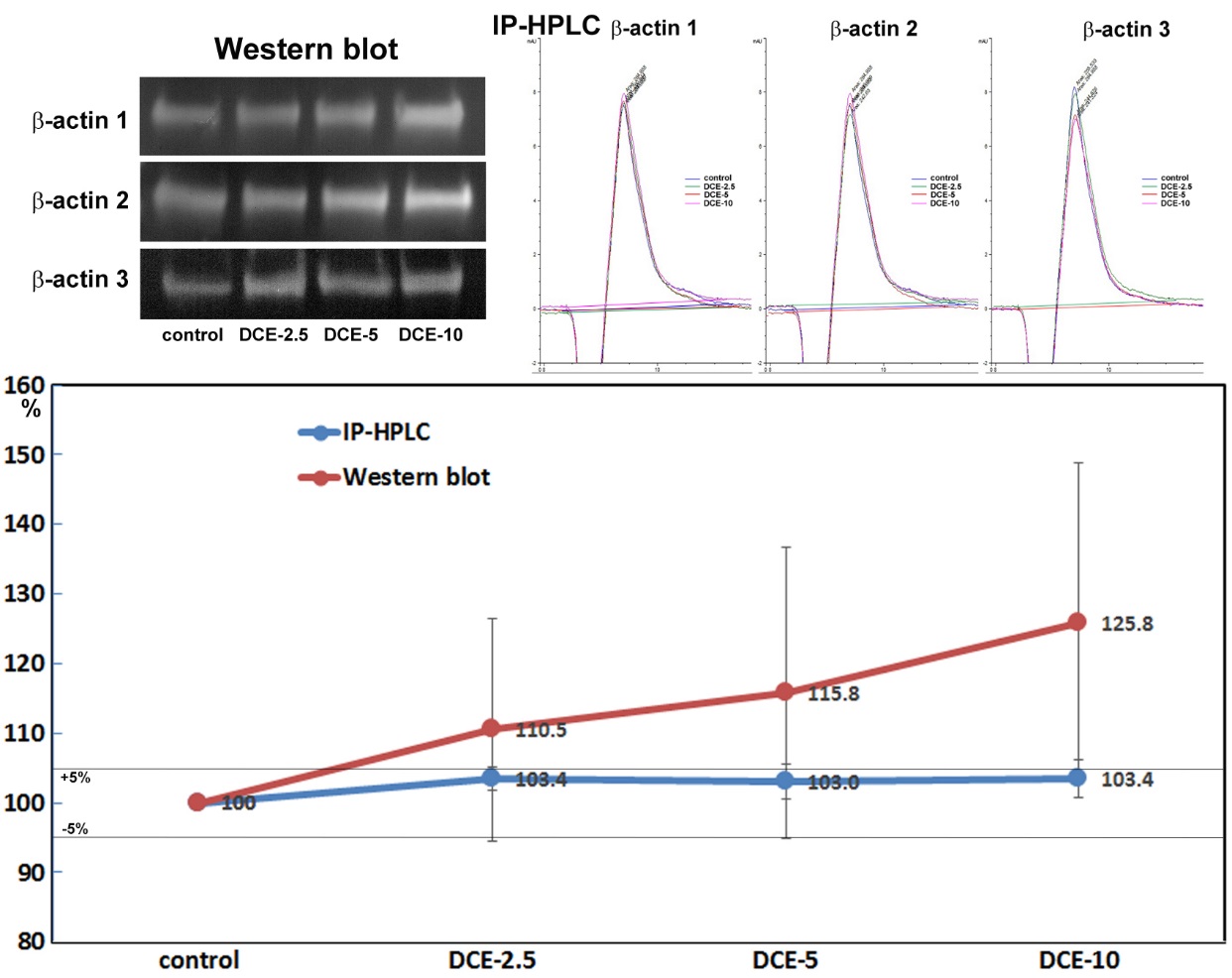


**Supplementary Figure 2.** β-Actin expression in DCE-treated RAW 264.7 cells was explored through western blot and IP-HPLC. Densitometry data of triplicated western blot (red line) showed big standard deviation (16.1 – 23.2 %), while triplicated IP-HPLC data (blue line) showed relatively small standard deviation (1.7 – 2.7%). Therefore, the latter was available to perform statistical analysis contrary to the former. These data were obtained from the previous study (Yoon, C.S., Kim, M.K., Kim, Y.S. & Lee, S.K. *In vitro* protein expression changes in RAW 264.7 cells and HUVECs treated with dialyzed coffee extract by immunoprecipitation high performance liquid chromatography. *Scientific reports* **8**, 13841 (2018))

*
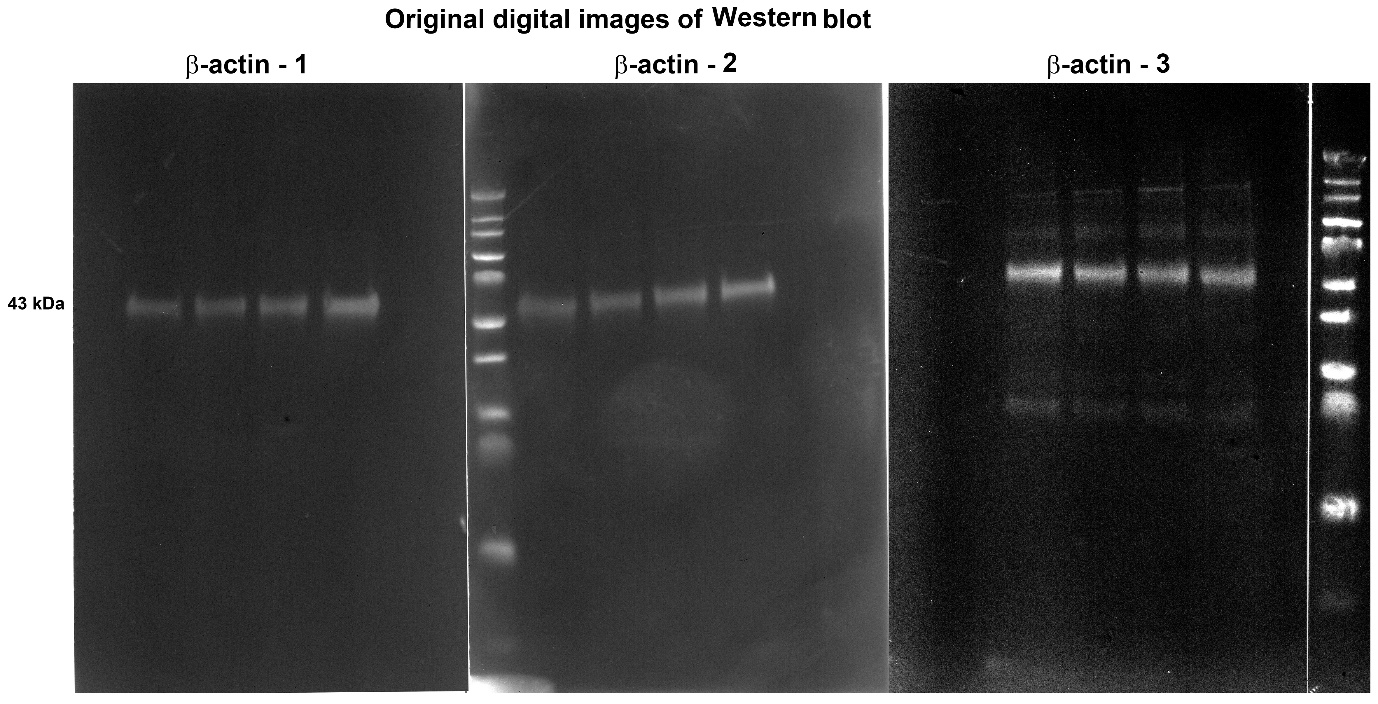
*
