# Supplementary material for: 4-hexylresorcinol-induced protein expression changes in human umbilical cord vein endothelial cells as determined by immunoprecipitation high-performance liquid chromatography
Source: PLoS One. 2020 Dec 15;15(12):e0243975. doi: 10.1371/journal.pone.0243975 (PMC7737996; doi:10.1371/journal.pone.0243975)
Supplement: S1 File — (PDF) [file pone.0243975.s003.pdf]

Original western blot gel image data

The selected protein expression levels of E-cadherin, VE-cadherin, TGF-β1, PARP-1, LC3, PERK, eIF2α, ATF4, and CHOP were examined by western blot for HUVECs treated with 10 μg/mL 4HR for 8, 16, or 24 h. The control was treated by normal saline only. The cells were collected with phosphate-buffered saline (PBS), treated with trypsin-ethylene-diamine-tetra-acetic acid (trypsin-EDTA) for 1 min and washed with PBS, and followed by cell lysis with ice-cold RIPA buffer (Sigma Aldrich, USA). The lysates were centrifuged at 12,000 g for 20 min at 4° C. The protein concentration of the supernatant was quantified using Bradford assay (BioRad, USA). Equal amounts (30 μg/lane) of sample proteins were separated by 8, 10, 15, or 20% sodium dodecyl sulfate-polyacrylamide gel electrophoresis (SDS-PAGE) in Tris-glycine SDS running buffer (25 mM Tris, 0.1% SDS, 0.2M glycine) to analyze the target proteins with protein marker. After the proteins were transferred from the gel to a nitrocellulose membrane, the membranes were blocked with 5% nonfat dry milk in TBST buffer (25 mM Tris-HCl, 150 mM NaCl, 0.1% Tween 20, pH 7.5) for 1 h. After being washed with TBST buffer three times, the membrane was incubated with each primary antibody (dilution ratio = 1:1000, the same antibody used in IP-HPLC) and horseradish peroxidase-conjugated secondary antibody for 1 h separately. Then, the protein bands were detected using an enhanced chemiluminescence system (Amersham Pharmacia Biotech, Piscataway, NJ, USA) according to the manufacturer's instructions, and digitally imaged using a ChemiDoc XRS system (Bio-Rad Laboratories, Hercules, CA, USA). The expression level of β-actin was used as an internal control to normalize the expression of the target proteins.

1. The images were directly cropped through ChemiDoc XRS system (Bio-Rad Laboratories, Hercules, CA, USA) during image analysis, and not changed through any other graphic program.
2. Every target protein was used proper SDS-PAGE gel concentration (8- 20%) depending on its molecular size.
3. The protein blot membrane was washed three times heavily, therefore, there showed conspicuous precursor proteins bands with no extra-bands.
4. Because the protein markers do not appear in the membrane, the molecular weight of each target protein was indicated in the figures.
5. Each protein was analyzed using separate SDS gel and membrane blot to prevent any contaminations or cross reactions. .

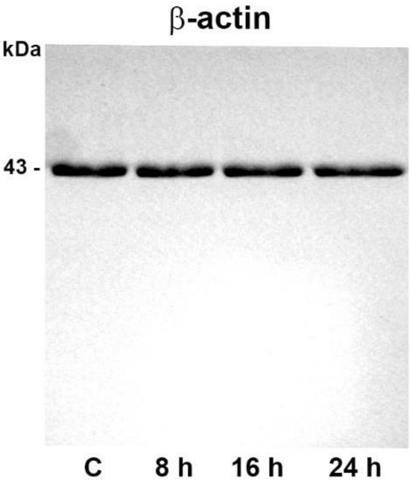

Date: August 17th-21st

15% SDS-PAGE gel

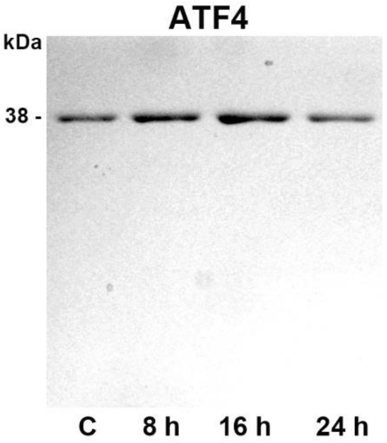

Date: August 17th-21st

15% SDS-PAGE gel

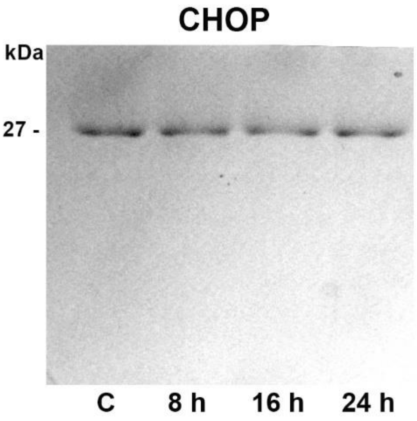

Date: August 17th-21st

15% SDS-PAGE gel

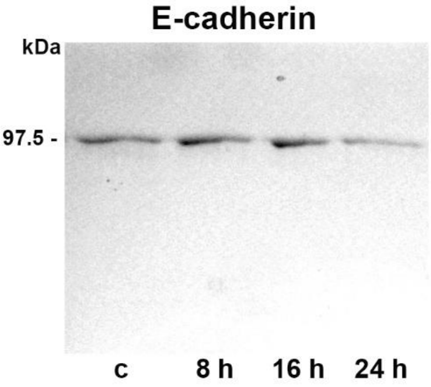

Date: August 17th-21st

10% SDS-PAGE gel

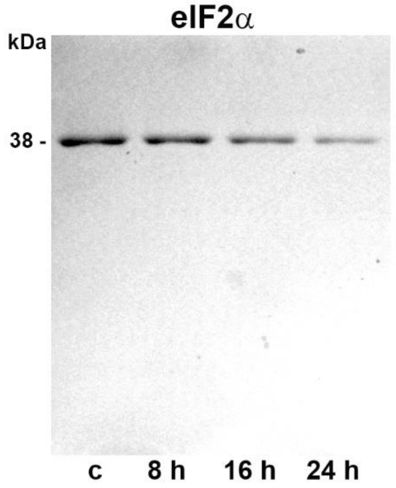

Date: August 17th-21st

15% SDS-PAGE gel

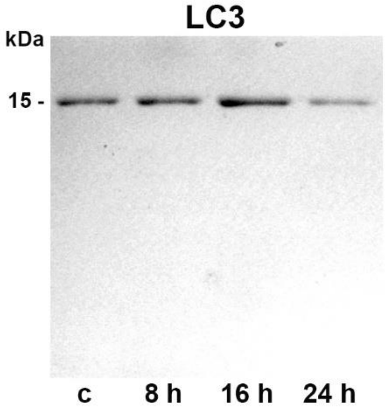

Date: August 24th-28st

20% SDS-PAGE gel

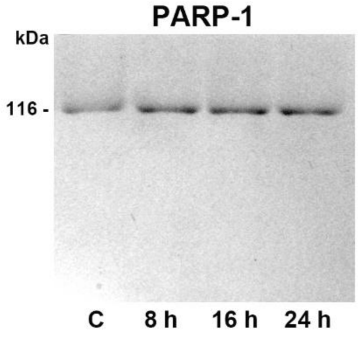

Date: August 24th-28st

10% SDS-PAGE gel

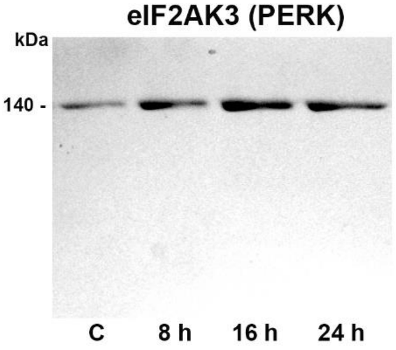

Date: August 24th-28st

8% SDS-PAGE gel

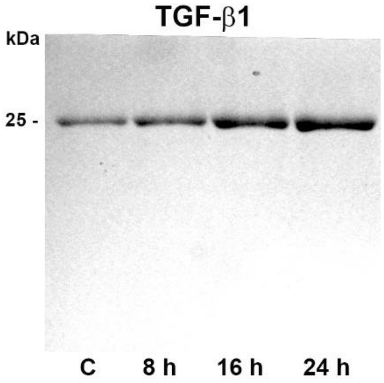

Date: August 24th-28st

15% SDS-PAGE gel

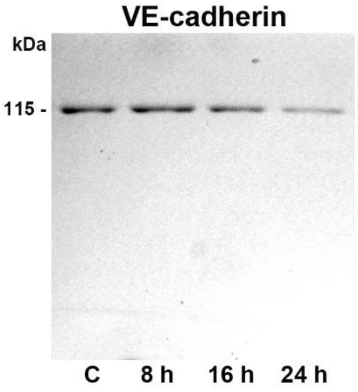

Date: August 24th-28st

8% SDS-PAGE gel

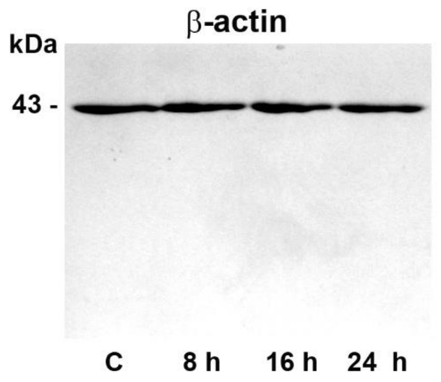

Date: November 9th-10th

8% SDS-PAGE gel

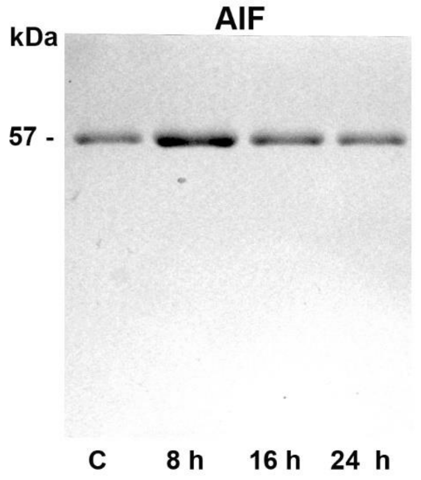

Date: November 9th-10th

8% SDS-PAGE gel

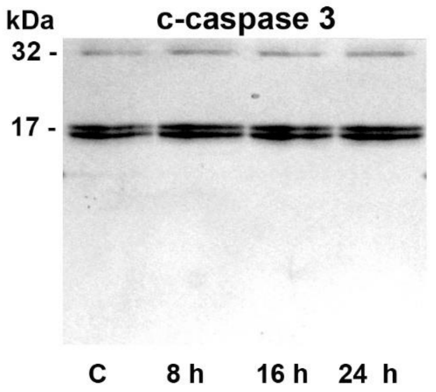

Date: November 9th-10th

8% SDS-PAGE gel

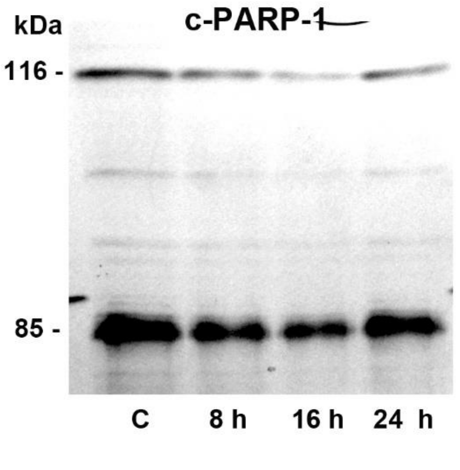

Date: November 9th-10th

10% SDS-PAGE gel
