# Supplementary material for: 4-hexylresorcinol-induced protein expression changes in human umbilical cord vein endothelial cells as determined by immunoprecipitation high-performance liquid chromatography
Source: PLoS One. 2020 Dec 15;15(12):e0243975. doi: 10.1371/journal.pone.0243975 (PMC7737996; doi:10.1371/journal.pone.0243975)
Supplement: Attachment — Submitted filename: PONE-D-20-02990 Review.pdf [file pone.0243975.s004.pdf]

PONE-D-20-02990 - Review

This study examines the effect of 4-hexylresorcinol (4HR) on human umbilical vein endothelial cells (HUVEC) protein and phospho-protein expression (227 in total) at three time points over a 24-hour period, using IP-HPLC. Protein expression in treated cells was compared to control (untreated) cells and significantly upregulated and downregulated proteins were identified. As these proteins belonged to specific functional groups, this allowed the authors to infer the impact of 4HR in modulators of various physiological processes, including angiogenesis, inflammation, etc. A similar study was previously performed in RAW 264.7 (virus-transformed macrophages), and the purpose of the current study was to investigate whether the effects in HUVEC are similar or different to those found in RAW 264.7 cells.

Although the study generated a number of interesting observations of potential physiological impact (e.g., upregulation of pro-angiogenic factors, changes indicative of potential growth inhibitory and apoptotic effects, effects on key inflammation mediators, etc.), there are some gaps in the study, which make the manuscript unacceptable at the present form. It is important to add that, if improved, the information gained with this study could be important to our understanding of the effects of 4HR in humans and how this could be exploited for improvements in the food and cosmetic industry (and even biomedicine). Below are my specific comments on this manuscript.

1. The authors mentioned that 4HR is added to foods, tooth paste, and cosmetics (Abstract and Introduction), but it is unclear how this investigation will help (or not) with continuing its application to these industries. In other words, I did not see a clear rationale for this study other than figuring out if the effects they were going to find were different in HUVEC as compared to the ones that were previously found in RAW 264.7 cells.

2. How does a 10 micrograms per mL concentration of 4HR compare to what a human being will be exposed to by tooth paste/cosmetic use or food consumption? Is this a physiologically relevant dose? This should have been addressed in the methods section.

3. The methods do not explain the IP-HPLC well, why expose the lysate to protein G/A sepharose before exposing to the antibody? Proteins in the lysates would not bind with high affinity to protein G/A, it is the antibodies that do. Perhaps there is a control step first to discard proteins that bind to protein G/A in absence of antibodies? I did not see that control… Steps of the procedure need to be explained better.

4. Apart from the analysis of the IP-HPLC results, there is not additional confirmation of the changes in proteins and phopho-proteins, which should have been done for at least a group of them (e.g., the angiogenesis mediators, or the growth factors).

5. The methods used, including statistics, were appropriate and the results are well described, but the concluding sentences interpreting the results for each of the results subsections (and reiterated in the discussion) are speculative and simplistic. it is impossible to affirm that 4HR modulates any cellular or physiological process (proliferation, apoptosis, angiogenesis, etc.) based only on the upregulation and downregulation of proteins. Further, some of the statements are contradictory. For instance, in lines 201-202 the authors say "These results suggest 4HR inhibits cell proliferation by downregulation of major proliferation-activating proteins…". Then in line 286, the authors say "These results indicate that 4HR

enhances cellular growth…". This does not make sense, as you can not inhibit proliferation and enhance cell growth at the same time; as far as I understand it proliferation and cell growth are the same thing.

6. To complement the two previous points, the study is lacking three key components:

a) Confirmatory data for the observed changes for at least some proteins of interest, in the form of Western blots, ELISA, Immunocytochemistry, immunoflouorescence, etc.

b) Confirmatory data that a cellular process is actually occurring. The authors mention in lines 591-592 that "some HUVECS conspicuously underwent apoptosis during 24 h of culture in the histological observation…", but did not show this data. Flow cytometry for Annexin V to show that there is indeed apoptosis, or a cell proliferation assay to show that there is inhibition of proliferation; or a cord formation assay to demonstrate pro-angiogenic effects on HUVEC, etc., will be helpful.

c) Proof that a given protein is actually responsible of a cellular or physiological process. For instance, if the increase in TGF-beta 1 and TGF-beta 3 are confirmed, and the authors are proposing that these are responsible for apoptosis, growth inhibition, increase in VEGF-A expression, etc.; then perhaps they should use a TGF-beta blocking antibody, a small molecule inhibitor of TGF-beta receptor type I/II, or a silencing RNA to block protein expression, and see what happens to apoptosis, VEGF, and/or cell proliferation, etc. The authors could focus on apoptosis for instance, and at least propose a model by which 4HR promotes HUVEC apoptosis. Next, the authors will need to then discuss why inducing endothelial cell apoptosis by these compounds might be detrimental (or not) to humans. Or if the decide to focus on the potential pro-angiogenic effects, then perhaps this compound might have some clinical application in cases when angiogenesis might be beneficial. The way the manuscript is written now it is impossible to see where this research can lead us to, and why it was important to do it. Although this journal does not assess the impact of the research, I believe it is important to highlight the relevance of the research.

7. The manuscript also contains inconsistencies that suggest a limited understanding of the cell system used and the mechanism of action and regulation of the proteins assessed. For instance, E-cadherin upregulation by 4HR treatment is one of the effects reported for Wnt/beta-catenin signalling mediators, but the study was done in HUVEC, which are endothelial cells and therefore expected to express VE-cadherin (the vascular type II cadherin) and not E-cadherin (the epithelial type I cadherin). Is this really E-cadherin what the authors identified? If it is indeed E-cadherin, then has it been reported that HUVEC express E-cadherin? Another reason why double-checking with Western blots would have been helpful. The authors proposed that upregulation of E-cadherin by 4HR is due to enhanced binding by beta-catenin (<10% upregulation at 8 h and < 10% downregulation at 24 h), which resulted in reduced nuclear translocation of the later. What about the Snail downregulation caused by 4HR as well (24.3% at 8 h)? Isn't Snail a transcription factor that represses cdh1? This component of the equation will have to be taken into consideration for result interpretation if the E-cadherin (or VE-cadherin) results are confirmed with another method.

8. Finally, it is recommended for the updated form of the manuscript that the results are presented in a more succinct manner and without any further discussion, and then discussed in the corresponding Discussion section. The Results section of the manuscript was very long and this will make it more reader friendly.
