# Supplementary material for: 4-hexylresorcinol-induced protein expression changes in human umbilical cord vein endothelial cells as determined by immunoprecipitation high-performance liquid chromatography
Source: PLoS One. 2020 Dec 15;15(12):e0243975. doi: 10.1371/journal.pone.0243975 (PMC7737996; doi:10.1371/journal.pone.0243975)
Supplement: Attachment — Submitted filename: PONE-D-20-02990 R2.pdf [file pone.0243975.s006.pdf]

PONE-D-20-02990 – Review of submission 2

This was a resubmission of a study that examines the effect of 4-hexylresorcinol (4HR) on human umbilical vein endothelial cells (HUVEC) protein and phospho-protein expression (227 in total) at three time points over a 24-hour period, using IP-HPLC. Protein expression in treated cells was compared to control (untreated) cells and significantly upregulated and downregulated proteins were identified. As these proteins belonged to specific functional groups, this allowed the authors to infer the impact of 4HR in modulators of various physiological processes, including angiogenesis, inflammation, etc. A similar study was previously performed in RAW 264.7 (virus-transformed macrophages), and the purpose of the current study was to investigate whether the effects in HUVEC are similar or different to those found in RAW 264.7 cells.

The study generated a number of interesting observations of potential physiological impact (e.g., upregulation of pro-angiogenic factors, changes indicative of potential growth inhibitory and apoptotic effects, effects on key inflammation mediators, etc.), but there were some gaps in the initial submission, which made the manuscript unacceptable at the time. I specifically raised 8 major points that needed to be addressed. Authors fully or partially addressed points 1, 3, 4, 5, 6, 7 and 8. However, there are still major and minor concerns that need to be addressed.

Major:

1. How does a 10 micrograms per mL concentration of 4HR compare to what a human being will be exposed to by tooth paste/cosmetic use or food consumption? Is this a physiologically relevant dose? This should have been addressed in the methods section. It was specified that it was a concentration which, when previously tested, showed "positive protein expression in different cells"; what does this mean? This need to be clarified in line 96 of the Material and Methods. In addition, the answer in relation to the physiological relevance of this dose is not convincing. It was argued that, although the dose employed in the present study is significantly larger that the dose achieved by cosmetic/food products used by humans, the effect in human can be cumulative. What is the concentration achieved in tissue in humans? I also disagree that a 24 hour treatment with a high dose (acute exposure) is similar to a cumulative effect of lower doses (chronic exposure). If there is some literature the authors could cite to address these persistent concerns, it will greatly enhance the meaning and applicability of their results.

2. The results of the immunocytochemistry of various molecules in Figures 2 and 3 show interesting and potentially relevant changes in subcellular localization of various proteins in HUVEC treated with 4HR, which become more obvious as the time increases. These were not mentioned or discussed. For instance, a change from predominantly nuclear to cytoplasmic seems to be happening for eIF2AK3 and LC3. Is this a valid observation? If this an artefact resulting from the lower magnification at which some images were taken in the 0h control as compared to the other time points? The magnification should be the same in all panels, and subcellular localization differences must be described in results and discussed later on.

3. Although the manuscript was reviewed and it has significantly improved, there are still some parts of the discussion that affirm mechanistic aspects that were not experimentally demonstrated. It is important to revise lines 632-634, 648-653, 654-655 to reflect the fact that

results are only suggestive of the mechanism/processes mentioned in those lines but they do not represent a proof that they are actually happening. All this manuscript presents are correlations; the demonstration of the involvement of signalling pathways in specific phenomena requires systematic functional assays that were not performed.

4. Please revise the discussion related to the meaning of Western blot analysis of PARP-1 and Immunocytochemistry of caspase-3 and PARP-1. Increase in these proteins do not necessarily reflect apoptosis. You should have determined their cleaved counterparts, which are the actual markers of apoptosis. In fact, when we have used an antibody that recognizes both the full-length and cleaved form of these proteins, what we observed was a concomitant reduction in the full-length as the levels of the cleaved form went up. The molecular weight of PARP-1 is not shown in the Western blot, but based on the description of the antibody used (Material and Methods section) what is shown by the blots in Fig.4 is an increase in full-length protein.

Minor:

The whole manuscript will benefit from a revision of English grammar and style. Below are some suggestions related to this, missing information, or misleading information.

<u>Abstract</u>

1. Line 30: change “than non-treated” to “as compared to non-treated”.
2. Line 31: Eliminate the word “Whereas” at the beginning of this sentence.
3. Line 34: switch “and had anti-inflammatory…” for “in a manner that suggest potential anti-inflammatory”.
4. Line 36: add the word “mediators” after “ER stresses”.
5. Lines 39-40: Eliminate “that is, HUVECs (endothelial cells) have strong regenerative potential for wound healing, while RAW 264.7 cells (macrophages) play a key role for inflammation”, and adjust punctuation accordingly.

<u>Introduction</u>

Needs a full revision of grammar and style.

<u>Materials and Methods</u>

1. Line 89: concentration of a few growth factors in media is missing.
2. Line 96: not sure what “positive protein expression” means; please explain.
3. Line 109: change the word “immunohistochemical” to “immunocytochemical (ICC)”, as you immunolabelled cells and not tissue. Make sure to change it in the results section and in the figure legends as well.
4. Lines 113 and 121: specify whether antibodies used for PARP-1 and caspase-3 recognize the full-length or the cleaved form; modify the description of corresponding results (Figs. 2 and 4) accordingly

<u>Results</u>

1. Line 256: “condensed” is not an appropriate description of the changes observed; please revise
2. Line 268: eliminate caspase-3 from the result description, as it was not tested by Western blotting. Also eliminate in Figure legend (line 278). Alternatively, if you have a blot for it, then include.
3. Fig. 4: Add MW of proteins to each blot.
4. Line 270-272: confusing sentence; please rewrite.

Discussion

1. Line 674: Not sure what the authors mean by “crosstalk between TGF-beta and SMAD signalling”. There is no crosstalk between TGF-beta and SMAD signalling. SMAD signalling is canonically activated by TGF-beta.
2. Line 703: switch “produce a strong angiogenic effect by upregulating” to “upregulated”.
3. Line 714: please revise grammar in the following sentence “although 4HR more upregulated some growth factors and stimulated downstream of RAS signaling in HUVECs than in RAW 264.7 cells”.
