# Supplementary material for: 4-hexylresorcinol-induced protein expression changes in human umbilical cord vein endothelial cells as determined by immunoprecipitation high-performance liquid chromatography
Source: PLoS One. 2020 Dec 15;15(12):e0243975. doi: 10.1371/journal.pone.0243975 (PMC7737996; doi:10.1371/journal.pone.0243975)
Supplement: Attachment — Submitted filename: Response to Reviewer comments-200906.docx [file pone.0243975.s005.docx]

**Dear Editor,**

We have done the major revision of this manuscript by including the new results of cell counting assay, immunohistochemistry, western blot, and additional IP-HPLC (Fig. 1-4 and Fig. 13 and 14). In the text, many sentences were corrected or rewritten according to the reviewer’s comments as follows;

**PONE-D-20-02990 - Review**

This study examines the effect of 4-hexylresorcinol (4HR) on human umbilical vein endothelial cells (HUVEC) protein and phospho-protein expression (227 in total) at three time points over a 24-hour period, using IP-HPLC. Protein expression in treated cells was compared to control (untreated) cells and significantly upregulated and downregulated proteins were identified. As these proteins belonged to specific functional groups, this allowed the authors to infer the impact of 4HR in modulators of various physiological processes, including angiogenesis, inflammation, etc. A similar study was previously performed in RAW 264.7 (virus-transformed macrophages), and the purpose of the current study was to investigate whether the effects in HUVEC are similar or different to those found in RAW 264.7 cells.

Although the study generated a number of interesting observations of potential physiological impact (e.g., upregulation of pro-angiogenic factors, changes indicative of potential growth inhibitory and apoptotic effects, effects on key inflammation mediators, etc.), there are some gaps in the study, which make the manuscript unacceptable at the present form. It is important to add that, if improved, the information gained with this study could be important to our understanding of the effects of 4HR in humans and how this could be exploited for improvements in the food and cosmetic industry (and even biomedicine).

**[Answer]**

Recently 4HR has been widely used in the food and cosmetic industry, therefore, the precise cellular effects induced by 4HR is necessary to know 4HR-induced physiological roles or hazard effect. In the previous study, RAW 264.7 cells which are derived from murine monocytes were explored, because they are essential defensing cells which initially engulf or immunologically react with 4HR molecules. And the present study explored HUVECs which play an important role for wound healing procedures. The minute amount of 4HR derived from tooth pastes, food additive, and cosmetic agents may directly affect macrophages (RAW 264.7 cells) and endothelial cells (HUVECs) *in vivo*. Therefore, we thought this study is helpful to understand the biological behavior of 4HR conveyed through food and cosmetics.

Below are my specific comments on this manuscript.

1. The authors mentioned that 4HR is added to foods, tooth paste, and cosmetics (Abstract and Introduction), but it is unclear how this investigation will help (or not) with continuing its application to these industries. In other words, I did not see a clear rationale for this study other than figuring out if the effects they were going to find were different in HUVEC as compared to the ones that were previously found in RAW 264.7 cells.

**[Answer]**

In order to make clear the rationale for this experiment, we added a description was added to explain why HUVECs were selected in this study in Introduction section as follow;

“As the angiogenesis and osteogenesis are important phenomena for tissue organogenesis, it is suggested HUVECs (endothelial cells), counterparts of RAW 264.7 cells (macrophages) in inflammation and wound healing, are also need to be explored for global protein expression changes induced by 4HR.” Line; 76-79

2. How does a 10 micrograms per mL concentration of 4HR compare to what a human being will be exposed to by tooth paste/cosmetic use or food consumption? Is this a physiologically relevant dose? This should have been addressed in the methods section.

**[Answer]**

In the previous studies, we explored 4HR effect on cells depending the concentration, 0.1, 1, 5, 10, 20, 50, 100 μg/mL, and then it was found that 10 μg/mL 4HR treatment showed consistent cleat positive results in different cells. Therefore, we choose 10 μg/mL concentration of 4HR treatment in this study. However, regarding the side effect of food additive and cosmetic agents containing 4HR, the absorbed amount of 4HR through gastrointestinal tract, mucosa, and skin might be much smaller than 10 μg/mL. However, there could appear an accumulated effect of 4HR when those agents are used for a longtime. In this study, we want to know unique cellular effect after 4HR treatment within 24 hours.

A sentence was added to define the reason to select a single concentration of 4HR, 10 μg/mL, as follow;

The concentration of 4HR treatment, 10 μg/mL, was a minimum effective dose determined through the previous studies, which consistently showed positive protein expressions in different cells [12, 13, 25-27]. Lines; 95-97

3. The methods do not explain the IP-HPLC well, why expose the lysate to protein G/A sepharose before exposing to the antibody? Proteins in the lysates would not bind with high affinity to protein G/A, it is the antibodies that do. Perhaps there is a control step first to discard proteins that bind to protein G/A in absence of antibodies? I did not see that control… Steps of the procedure need to be explained better.

**[Answer]**

There must be occurred something during manuscript edition, and it was clearly a mistyping, therefore, we correct the sentences as follow in the text;

“Protein A/G agarose beads column (Amicogen, Jinju, Korea) were separately pre-incubated with 1 μg of 234 different antisera shown in Table 1 in Tris-HCl buffer (pH 7.5, 0.1% Tween 20) at room temperature for 1 h. The supernatant of antibody-incubated column was removed, and then followed by immunoprecipitation procedures.” Lines; 140-143

4. Apart from the analysis of the IP-HPLC results, there is not additional confirmation of the changes in proteins and phopho-proteins, which should have been done for at least a group of them (e.g., the angiogenesis mediators, or the growth factors).

**[Answer]**

In this study, several phosphor-proteins were used to compared with the expression of precursor or non-phospor proteins; p-SMAD4, p-JNK, p-ERK-1, p-p38, pPKC1α, pAKT1/2/3, and p-VEGFR2, and at this time, p-eIF2AK3, and p-eIF2α were added in IP-HPLC analysis. Unfortunately, those antibodies were used in the additional experiments of western blot and immunohistochemistry. Therefore, we tried to find some expression changes between phosphor-protein and non-phosphor-proteins in IP-HPLC analysis in this study.

5. The methods used, including statistics, were appropriate and the results are well described, but the concluding sentences interpreting the results for each of the results subsections (and reiterated in the discussion) are speculative and simplistic. it is impossible to affirm that 4HR modulates any cellular or physiological process (proliferation, apoptosis, angiogenesis, etc.) based only on the upregulation and downregulation of proteins. Further, some of the statements are contradictory. For instance, in lines 201- 202 the authors say “These results suggest 4HR inhibits cell proliferation by downregulation of major proliferation-activating proteins…”. Then in line 286, the authors say “These results indicate that 4HR 2 enhances cellular growth…”. This does not make sense, as you can not inhibit proliferation and enhance cell growth at the same time; as far as I understand it proliferation and cell growth are the same thing.

**[Answer]**

The contradictory findings in 4HR-treated HUVECs were compared with the protein expressions in 4HR-treated RAW 264.7 cells in this study. We made a discussion about these contradictory events in discussion section as follow;

“However, the coincident downregulation of proliferation and upregulation of growth by 4HR may be contradictory in cells, and then, 4HR usually upregulated reactive growth factors including TGF-βs, HGFα, IGF-1, and HER1 instead of major growth factors including FGF-1, -2, GH, GHRH, PDGF-A, and CTGF, while suppressed proliferation by upregulating different mitosis and cyclin-related proteins. Therefore, it is believed that 4HR-induced effects on HUVECs and RAW 264.7 cells are still safe and well balanced by cellular homeostasis.” Line; 633-638

And in order to reduce a confusion from the discrepancies in global protein expressions, two diagrams were made to illustrated the overall interactions between signaling pathways (Fig. 13 and 14)

6. To complement the two previous points, the study is lacking three key components:

a) Confirmatory data for the observed changes for at least some proteins of interest, in the form of Western blots, ELISA, Immunocytochemistry, immunoflouorescence, etc.

**[Answer]**

Immunohistochemistry (IHC) and western blot analysis were performed to confirm the expressions of E-cadherin and VE-cadherin for endothelial cell differentiation, the expressions of TGF-β1, caspase 3, and PARP-1 for cellular apoptosis, and the expressions of PERK, eIF2α, ATF4, ATF6, CHOP, and LC3 for ER stresses in 4HR-treated HUVECs.

IHC and western blot results were added into Result section with Figures 2-4 in the text.

b) Confirmatory data that a cellular process is actually occurring. The authors mention in lines 591-592 that "some HUVECS conspicuously underwent apoptosis during 24 h of culture in the histological observation…", but did not show this data. Flow cytometry for Annexin V to show that there is indeed apoptosis, or a cell proliferation assay to show that there is inhibition of proliferation; or a cord formation assay to demonstrate pro-angiogenic effects on HUVEC, etc., will be helpful.

**[Answer]**

Because highly adherent cells like HUVECs are usually difficult to manipulate for flow-cytometry analysis, we have done direct cell counting on the surfaces of culture dishes to define the proliferation activity if 4HR-treated HUVECs instead of flow-cytometry at this time. The result is illustrated in Fig. 1.

And to confirm the apoptosis in 4HR-treated HUVECs, the immunohistochemical observation with caspase 3 and PARP-1 antibodies and Western blot analysis with PARP-1 and TGF-β1 were performed and illustrated in Figures 2-4.

c) Proof that a given protein is actually responsible of a cellular or physiological process. For instance, if the increase in TGF-beta 1 and TGF-beta 3 are confirmed, and the authors are proposing that these are responsible for apoptosis, growth inhibition, increase in VEGF-A expression, etc.; then perhaps they should use a TGF-beta blocking antibody, a small molecule inhibitor of TGF-beta receptor type I/II, or a silencing RNA to block protein expression, and see what happens to apoptosis, VEGF, and/or cell proliferation, etc. The authors could focus on apoptosis for instance, and at least propose a model by which 4HR promotes HUVEC apoptosis. Next, the authors will need to then discuss why inducing endothelial cell apoptosis by these compounds might be detrimental (or not) to humans. Or if the decide to focus on the potential pro-angiogenic effects, then perhaps this compound might have some clinical application in cases when angiogenesis might be beneficial. The way the manuscript is written now it is impossible to see where this research can lead us to, and why it was important to do it. Although this journal does not assess the impact of the research, I believe it is important to highlight the relevance of the research.

**[Answer]**

In the serial studies of 4HR effect on HUVECs done in our laboratory, the other paper has already reported the angiogenesis induced via TGF-β signaling assessed by Western blot using TGF-β1 inhibitor, siRNA of TGF-β1, TGF-β receptor inhibitor (Increased Level of Vascular Endothelial Growth Factors by 4-hexylresorcinol is Mediated by Transforming Growth Factor-beta1 and Accelerates Capillary Regeneration in the Burns in Diabetic Animals. International journal of molecular sciences. 2020;21(10)). The present results of TGF-β1 signaling are almost identical with the above paper results.

7. The manuscript also contains inconsistencies that suggest a limited understanding of the cell system used and the mechanism of action and regulation of the proteins assessed. For instance, E-cadherin upregulation by 4HR treatment is one of the effects reported for Wnt/beta-catenin signalling mediators, but the study was done in HUVEC, which are endothelial cells and therefore expected to express VEcadherin (the vascular type II cadherin) and not E-cadherin (the epithelial type I cadherin). Is this really E-cadherin what the authors identified? If it is indeed E-cadherin, then has it been reported that HUVEC express E-cadherin? Another reason why double-checking with Western blots would have been helpful. The authors proposed that upregulation of E-cadherin by 4HR is due to enhanced binding by betacatenin (< 10% downregulation at 24 h), which resulted in reduced nuclear translocation of the later. What about the Snail downregulation caused by 4HR as well (24.3% at 8 h)? Isn’t Snail a transcription factor that represses cdh1? This component of the equation will have to be taken into consideration for result interpretation if the E-cadherin (or VE-cadherin) results are confirmed with another method.

**[Answer]**

In order to correct some inconsistencies of description, the diagrams of Fig. 13 and 14 were added, and many sentences were rewritten to go straight for the experimental goals.

And IHC staining and Western blot were performed to confirm the expressions of E-cadherin and VE-cadherin at this time. We found both of E-cadherin and VE-cadherin were conspicuously expressed in HUVECs and showed increased expression in 4HR-treated HUVECs. In the immunostainings of E-cadherin and VE-cadherin, there appeared positive reaction in HUVECs after 4HR treatment, and Western blot of E-cadherin and VE-cadherin disclosed slight increase in 4HR-treated HUVECs at 8 and 16 h (Fig. 2-4)

8. Finally, it is recommended for the updated form of the manuscript that the results are presented in a more succinct manner and without any further discussion, and then discussed in the corresponding Discussion section. The Results section of the manuscript was very long and this will make it more reader friendly.

**[Answer]**

All short comments in Result section were deleted, and these were discussed in Discussion section as the reviewer suggested. And the irrelevant descriptions were all removed.

Thank you very much for your kind comments.
