# Supplementary material for: 4-hexylresorcinol-induced protein expression changes in human umbilical cord vein endothelial cells as determined by immunoprecipitation high-performance liquid chromatography
Source: PLoS One. 2020 Dec 15;15(12):e0243975. doi: 10.1371/journal.pone.0243975 (PMC7737996; doi:10.1371/journal.pone.0243975)
Supplement: Attachment — Submitted filename: Response to Reviewer comments-201115.docx [file pone.0243975.s007.docx]

**PONE-D-20-02990R1
4-hexylresorcinol-induced protein expression changes in human umbilical cord vein endothelial cells as determined by immunoprecipitation high performance liquid chromatography
PLOS ONE**

Reviewers' comments:

Reviewer #1: This was a resubmission of a study that examines the effect of 4-hexylresorcinol (4HR) on human umbilical vein endothelial cells (HUVEC) protein and phospho-protein expression (227 in total) at three time points over a 24-hour period, using IP-HPLC. Protein expression in treated cells was compared to control (untreated) cells and significantly upregulated and downregulated proteins were identified. As these proteins belonged to specific functional groups, this allowed the authors to infer the impact of 4HR in modulators of various physiological processes, including angiogenesis, inflammation, etc. A similar study was previously performed in RAW 264.7 (virus-transformed macrophages), and the purpose of the current study was to investigate whether the effects in HUVEC are similar or different to those found in RAW 264.7 cells.

The study generated a number of interesting observations of potential physiological impact (e.g., upregulation of pro-angiogenic factors, changes indicative of potential growth inhibitory and apoptotic effects, effects on key inflammation mediators, etc.), but there were some gaps in the initial submission, which made the manuscript unacceptable at the time. I specifically raised 8 major points that needed to be addressed. Authors fully or partially addressed points 1, 3, 4, 5, 6, 7 and 8. However, there are still major and minor concerns that need to be addressed.

Major:

1. How does a 10 micrograms per mL concentration of 4HR compare to what a human being will be exposed to by tooth paste/cosmetic use or food consumption? Is this a physiologically relevant dose? This should have been addressed in the methods section. It was specified that it was a concentration which, when previously tested, showed “positive protein expression in different cells”; what does this mean? This need to be clarified in line 96 of the Material and Methods. In addition, the answer in relation to the physiological relevance of this dose is not convincing. It was argued that, although the dose employed in the present study is significantly larger that the dose achieved by cosmetic/food products used by humans, the effect in human can be cumulative. What is the concentration achieved in tissue in humans? I also disagree that a 24 hour treatment with a high dose (acute exposure) is similar to a cumulative effect of lower doses (chronic exposure). If there is some literature the authors could cite to address these persistent concerns, it will greatly enhance the meaning and applicability of their results.

**[Answer]**

Several studies performed using different level of 4HR dose in animals (dogs, rats, mice, and cats) were cited in this text, and explained why the present study selected the dose of 10 μg/mL and performed the culture experiment for 24 h. The general animal experiments for 4HR effects were described in introduction section (line 58-79) and their dose-dependent treatments were described in materials and methods section (line 119-124).

Line 58-79: Under a two-year gavage study conducted by administering 0, 62.5 or 125 mg/kg (0, 62.5 or 125 μg/g) to groups of 50 F344/N rats and 50 B6C3F1 mice of each sex, five days per week, there was no significant differences in survival and no evidence of carcinogenic activity [9]. The oral LD_50_ of 4HR was 550 mg/kg body weight in rat [10, 11], 475 mg/kg in Guinea-pig [12], approximately 750 mg/kg in rabbit [12], and 200-1000 mg/kg in mice (subcutaneous injection with 5% 4HR in olive oil; 750-1000 mg/kg, intraperitoneal injection with 5% 4HR in olive oil; 200 mg/kg, with 1% 4HR aqueous emulsion; 300 mg/kg) [11]. The probable oral LD_50_ of 4-hexylresorcinol in humans has been estimated to be between 500 and 5000 mg/kg [13]. These data indicate 4 HR may have relatively wide range of applicable dose in animals and human, and that the dose used in this study, 10 μg/mL, is within a safe range and might be free of toxic chemical hazard.

4HR is excreted via the urine mainly in the form of an ethereal sulfate conjugate [14]. In the animal experiments [15], dogs were given single doses of 1 or 3 g 4HR (equivalent to 100 or 300 mg/kg body weight) as crystals in gelatin capsules or as a solution in olive oil, and its excretion in urine and feces was monitored. After administrating 1 g crystalline compound, 29% of the dose was detected in urine and 67% in feces. When the dose was increased to 3 g, 17% and 73% was excreted in urine and feces, respectively. Urinary excretion was rapid, mainly in the first 6 h, and levels were virtually undetectable 12 h after the lower dose and 24-36 h following the higher dose. When 4HR was administered in olive oil, a dose of 1 g resulted in 17% and 76% was excreted in urine and feces, respectively, while 3 g, 10% and 80% was excreted in urine and feces, respectively. When two men received doses of 1 g 4HR, an average of 18% of the dose was recovered in urine within the first 12 h. Thereafter, the compound was not detected in urine samples. Fecal excretion accounted for 64% of the dose [16]. These results suggest the metabolic degradation of 4HR is vigorous for 6 h and persists until 24 h. Therefore, the present study performed 4HR treatment for 24 h in cell culture experiment.

Lie 119-124: If 20% of 4HR administered orally was absorbed in different animals, the cellular level dose of 4HR was calculated to be 20 or 60 mg/kg in dog [15], 12 mg/kg in cat [33], 12.5 or 25 mg/kg in rat [9], and 25, 50 or 100 mg/kg in mice [9]. 4HR is commonly employed in 1:1000 solution or [glycerite](https://pubchem.ncbi.nlm.nih.gov/compound/glycerite) (0.01%, 194 μg/mL) in mouthwashes or pharyngeal antiseptic preparation [34]. Therefore, the dose used in the present study, 10 μg/mL, is safe within the experimental range, and the previous studies used the same dose of 4HR had showed characteristic protein expression in cell culture [20, 21, 35-37]. Cultured cells were harvested with protein lysis buffer (PRO-PREP^TM^, iNtRON Biotechnology, Daejeon, Korea) in ice, and immediately preserved at -70°C until required.

2. The results of the immunocytochemistry of various molecules in Figures 2 and 3 show interesting and potentially relevant changes in subcellular localization of various proteins in HUVEC treated with 4HR, which become more obvious as the time increases. These were not mentioned or discussed. For instance, a change from predominantly nuclear to cytoplasmic seems to be happening for eIF2AK3 and LC3. Is this a valid observation? If this an artefact resulting from the lower magnification at which some images were taken in the 0h control as compared to the other time points? The magnification should be the same in all panels, and subcellular localization differences must be described in results and discussed later on.

**[Answer]**

The immunocytochemcal results of eIF2AK3, eIF2α, ATF4, LC3, lysozyme, and PARP-1 were described in results section (287-293 and 299-311), and also discussed them in discussion section (line 745-759).

All the figure panels were taken in the same magnification, which were indicated with bar ruler at lower right corner of each figure.

Line 287-293 and 299-311: Caspase 3, an apoptosis executing enzyme, was strongly positive in 4HR-treated cells compared to the untreated control cells, and its immunoreaction was localized at the nuclei (Fig. 2D). PARP-1, a highly error-prone DNA repair pathway microhomolgy-mediated end joining enzyme, was usually positive in the nuclei of untreated control cells, but its immunoreaction was increased gradually in the cytoplasm of 4HR-treated cells at 8, 16, and 24 h (Fig. 2E). Lysozyme, a cationic muiramidase, was diffusely positive in the cytoplasm of untreated control cells, but its immunoreaction was localized at the nuclei of 4HR-treated cells at 8, 16, and 24 h (Fig. 2F).

The immunoreaction of eIF2AK3, a protein kinase R (PKR)-like endoplasmic reticulum kinase (PERK) that can inactivate eIF2α, increased gradually in 4HR-treated cells at 8, 16, and 24 h compared to the untreated control cells. eIF2AK3 was diffusely localized at the cytoplasm and nuclei of cells (Fig. 3A). eIF2α, a regulator of global translation in response to cellular stress, was weakly positive in the untreated control cells, but it increased gradually in 4HR-treated cells at 8, 16, and 24 h (Fig. 3B). ATF4, a master transcription factor during the integrated stress response, was weakly positive in the untreated control cells but became strongly positive and localized at the nuclei of 4HR-treated cells at 8, 16, and 24 h (Fig. 3C).

GADD153 (CHOP, DDIT3), a DNA damage-inducible transcript 3 pro-apoptotic transcription factor, was weakly positive in the untreated control cells, but its immunoreaction increased slightly in the nuclei of 4HR-treated cells at 8, 16, and 24 h (Fig. 3D). LC3, a biomarker of autophagosomes, was strongly positive in the nuclei but weak in the cytoplasm of the untreated cells. The immunoreaction of LC3 was observed in both the cytoplasm and nuclei of HUVECs, and was higher in 4HR-treated cells at 8, 16, and 24 h, and localized at the cytoplasm of cells but sparse in the nuclei (Fig. 3E).

Line 745-759: The histology examination of the cells spread over the surface of the culture slide dish revealed many small vacuoles in the cytoplasm of 4HR-treated HUVECs compared to the untreated controls. The small vacuoles gradually occupied the entire cytoplasm of HUVECs, which were strongly positive for LC3 but weakly positive for lysozyme in ICC staining. Therefore, it was assumed that the small vacuoles belong to autophages, resulting from ER stresses induced by 4HR. This assumption was investigated with IP-HPLC, ICC, and western blot analyses. In the IP-HPLC, eIF2AK3, a protein kinase R-like endoplasmic reticulum kinase (PERK), and p-eIF2AK3 were upregulated simultaneously in 8, 16, and 24 h. In contrast, eIF2α was downregulated with overexpression of p-eIF2α in 16 and 24 h. Transcription factors responding to ER stresses, ATF4 and ATF6 were consistently upregulated, but a DNA damage-inducible pro-apoptotic transcription factor, GADD153 was downregulated at 8, 16, and 24 h. These results suggest that eIF2AK3 was active and rapidly phosphorylated into p-eIF2AK3 which subsequently inactivated eIF2α by phosphorylating the alpha subunit of eIF2α, resulting in the repression of global protein synthesis in 4HR-treated cells. The consistent upregulation of ATF4 and ATF6 and the downregulation of GADD153 might rescue 4HR-treated HUVECs from apoptotic damage, as well as the coincident upregulation of LC3 has a protective role of autophagosomes which were found in 4HR-treated cells at 8, 16, and 24 h.

3. Although the manuscript was reviewed and it has significantly improved, there are still some parts of the discussion that affirm mechanistic aspects that were not experimentally demonstrated. It is important to revise lines 632-634, 648-653, 654-655 to reflect the fact that results are only suggestive of the mechanism/processes mentioned in those lines but they do not represent a proof that they are actually happening. All this manuscript presents are correlations; the demonstration of the involvement of signalling pathways in specific phenomena requires systematic functional assays that were not performed.

**[Answer]**

The sentences were corrected as indicated by the reviewer’s comment in the manner to describe simple phenomena in the protein expressions rather than to specify their signaling pathways throughout the text.

4. Please revise the discussion related to the meaning of Western blot analysis of PARP-1 and Immunocytochemistry of caspase-3 and PARP-1. Increase in these proteins do not necessarily reflect apoptosis. You should have determined their cleaved counterparts, which are the actual markers of apoptosis. In fact, when we have used an antibody that recognizes both the full-length and cleaved form of these proteins, what we observed was a concomitant reduction in the full-length as the levels of the cleaved form went up. The molecular weight of PARP-1 is not shown in the Western blot, but based on the description of the antibody used (Material and Methods section) what is shown by the blots in Fig.4 is an increase in full-length protein.

**[Answer]**

Regarding the results of western blot, the data of c-caspase 3, c-PARP-1, and AIF were added, and the possible evidence of apoptosis was discussed with the elevation of c-PARP-1, a hallmark of apoptosis as an inhibited cleaved form after over-activation of PARP-1, during 8 – 24 h after 4HR treatment, and consistent increase of PARP-1, an effector enzyme for DNA repair and apoptosis in 8, 16, and 24 h (line 724-744).

The molecular weight of each protein was marked on the panel of western blot. And the antigenic epitopes of caspase 3, c-caspase 3, PARP-1, and PARP-1 were described in materials and methods section.

Minor:

The whole manuscript will benefit from a revision of English grammar and style. Below are some suggestions related to this, missing information, or misleading information.

Abstract

1. Line 30: change “than non-treated” to “as compared to non-treated”.

**[Answer]**

It was corrected as the reviewer indicated.

2. Line 31: Eliminate the word “Whereas” at the beginning of this sentence.

**[Answer]**

It was deleted as the reviewer indicated.

3. Line 34: switch “and had anti-inflammatory…” for “in a manner that suggest potential anti-inflammatory”.

**[Answer]**

It was corrected as the reviewer indicated.

4. Line 36: add the word “mediators” after “ER stresses”.

**[Answer]**

It was corrected as the reviewer indicated.

5. Lines 39-40: Eliminate “that is, HUVECs (endothelial cells) have strong regenerative potential for wound healing, while RAW 264.7 cells (macrophages) play a key role for inflammation”, and adjust punctuation accordingly.

**[Answer]**

It was deleted as the reviewer indicated, and adjusted its punctuation.

Introduction

Needs a full revision of grammar and style.

**[Answer]**

The whole text including introduction was re-revised by a professional personnel in Nurisco English Editing Company in Korea.

Materials and Methods

1. Line 89: concentration of a few growth factors in media is missing.

**[Answer]**

The concentrations of those growth factors were inserted in the text.

2. Line 96: not sure what “positive protein expression” means; please explain.

**[Answer]**

The sentence was rewritten in an appropriate manner to keep from making a confusion.

Line 122-124: Therefore, the dose used in the present study, 10 μg/mL, is safe within the experimental range, and the previous studies used the same dose of 4HR had showed characteristic protein expression in cell culture [20, 21, 35-37].

3. Line 109: change the word “immunohistochemical” to “immunocytochemical (ICC)”, as you immunolabelled cells and not tissue. Make sure to change it in the results section and in the figure legends as well.

**[Answer]**

The word “immunohistochemical” was changed into “immunocytochemical (ICC)” in the whole text.

4. Lines 113 and 121: specify whether antibodies used for PARP-1 and caspase-3 recognize the full-length or the cleaved form; modify the description of corresponding results (Figs. 2 and 4) accordingly

**[Answer]**

The full-length or the cleaved form was described for PARP-1 and c-caspase 3 in the text.

Results

1. Line 256: “condensed” is not an appropriate description of the changes observed; please revise

**[Answer]**

The word “condensed” was changed into “localized at” in the text.

2. Line 268: eliminate caspase-3 from the result description, as it was not tested by Western blotting. Also eliminate in Figure legend (line 278). Alternatively, if you have a blot for it, then include.

**[Answer]**

Instead of caspase 3, c-caspase 3 was tested by western blot and its result was described in the text.

3. Fig. 4: Add MW of proteins to each blot.

**[Answer]**

Molecular weights of proteins were marked on the western blot figure.

4. Line 270-272: confusing sentence; please rewrite.

**[Answer]**

Line 270-272 was rewritten as follows;

Line 323-326: eIF2AK3 (PERK), eIF2α, ATF4, and GADD153, contributing eIF2AK3/eIF2α/ATF4/GADD153 signaling for ER stresses, increased or decreased variably, and the expression of eIF2AK3 and ATF4 increased significantly at 8, 16, and 24 h after 4HR treatment. GADD153 expression changed minimally after the 4HR treatment, while eIF2α expression decreased slightly.

Discussion

1. Line 674: Not sure what the authors mean by “crosstalk between TGF-beta and SMAD signaling”.

There is no crosstalk between TGF-beta and SMAD signaling. SMAD signaling is canonically activated by TGF-beta.

**[Answer]**

The description about TGF-β1 and SMAD signaling was rewritten as follows;

Line 736-744: This 4HR-induced cellular apoptosis would be progressive and involved in the alternative activation of NFkB signaling or the compensatory stimulation of TGF-βs production. In the present study, 4HR-treated HUVECs strongly expressed TGF-β1, -β2, and -β3 despite the consistent downregulation of FGF-1, FGF-2, FGF-7, GH, GHRH, PDGF-A, and c-erbB-2 (HER2). The dominant expression of TGF-β1, -β2, and β3 may lead to activation of the SMAD2/3/SMAD4 pathway, resulting in the transcription of the target genes (e.g., VEGFs and BMPs) and the activations of RAF-B/ERK and p38 signaling [21, 22, 50, 51]. In the present study, these TGF-β signaling cascades were upregulated markedly by 4HR in HUVECs, which increased the expression of RAF-B, SMADs, ERK-1, p38, VEGFs, and BMP-2. Therefore, HUVECs have strong regenerative properties to react with 4HR by upregulating TGF-βs.

2. Line 703: switch “produce a strong angiogenic effect by upregulating” to “upregulated”.

**[Answer]**

This line was corrected as the reviewer indicated.

3. Line 714: please revise grammar in the following sentence “although 4HR more upregulated some growth factors and stimulated downstream of RAS signaling in HUVECs than in RAW 264.7 cells”.

**[Answer]**

This sentence was corrected as follows;

Line 792-793: but 4HR upregulated some growth factors and some downstream proteins of RAS signaling more in HUVECs than in RAW 264.7 cells.

I am greatly appreciated your kind correction by sharing your precious time.

Thank you very much, again.
